# Generation of a hTERT-Immortalized Human Sertoli Cell Model to Study Transporter Dynamics at the Blood-Testis Barrier

**DOI:** 10.3390/pharmaceutics12111005

**Published:** 2020-10-22

**Authors:** Raymond K. Hau, Siennah R. Miller, Stephen H. Wright, Nathan J. Cherrington

**Affiliations:** 1 College of Pharmacy, Department of Pharmacology & Toxicology, University of Arizona, Tucson, AZ 85719, USA; hau@pharmacy.arizona.edu (R.K.H.); smiller@pharmacy.arizona.edu (S.R.M.); 2 College of Medicine, Department of Physiology, University of Arizona, Tucson, AZ 85719, USA

**Keywords:** cell immortalization, sertoli cell, blood–testis barrier, testes, xenobiotic transporter, drug disposition, nucleoside transport, uridine, male contraceptive, antiviral

## Abstract

The blood-testis barrier (BTB) formed by adjacent Sertoli cells (SCs) limits the entry of many chemicals into seminiferous tubules. Differences in rodent and human substrate-transporter selectivity or kinetics can misrepresent conclusions drawn using rodent in vitro models. Therefore, human in vitro models are preferable when studying transporter dynamics at the BTB. This study describes a hTERT-immortalized human SC line (hT-SerC) with significantly increased replication capacity and minor phenotypic alterations compared to primary human SCs. Notably, hT-SerCs retained similar morphology and minimal changes to mRNA expression of several common SC genes, including AR and FSHR. The mRNA expression of most xenobiotic transporters was within the 2-fold difference threshold in RT-qPCR analysis with some exceptions (OAT3, OCT3, OCTN1, OATP3A1, OATP4A1, ENT1, and ENT2). Functional analysis of the equilibrative nucleoside transporters (ENTs) revealed that primary human SCs and hT-SerCs predominantly express ENT1 with minimal ENT2 expression at the plasma membrane. ENT1-mediated uptake of [^3^H] uridine was linear over 10 min and inhibited by NBMPR with an IC_50_ value of 1.35 ± 0.37 nM. These results demonstrate that hT-SerCs can functionally model elements of transport across the human BTB, potentially leading to identification of other transport pathways for xenobiotics, and will guide drug discovery efforts in developing effective BTB-permeable compounds.

## 1. Introduction

Polarized Sertoli cells (SCs) are the fundamental epithelial cells that line the seminiferous tubules of the testes and are essential for supporting and protecting developing germ cells. These cells are commonly referred to as the “nurse” cells of the testes because they are responsible for regulating the growth and development of germ cells by providing nutrients or phagocytosing apoptotic cells [1,2]. SCs express and secrete a number of growth factors and cytokines such as bone morphogenic protein 4 (BMP4), glial cell line-derived neurotrophic factor (GDNF), and fibroblast growth factor 2 (FGF2) to regulate self-renewal or germ cell development. Other proteins, such as SRY-box transcription factor 9 (SOX9) and follicle-stimulating hormone receptor (FSHR), are exclusively expressed by SCs and are essential for SC differentiation and function, respectively [3,4,5].

Adjacent SCs express junctional proteins that congregate and form the physical blood-testis barrier (BTB), including the tight junction proteins claudin-11 (CLDN11), occludin (OCLN), and tight junction protein-1/zonula occludens-1 (TJP1/ZO-1) [6,7]. The BTB acts as a physical, semi-selective barrier that regulates the movement of endogenous and exogenous substances from the circulatory or lymphatic systems into the adluminal environment of the seminiferous tubule [8,9,10]. Although the BTB is beneficial for germ cell development, it also impedes the penetration and accumulation of drugs intended to treat male genital tract (MGT) disorders from reaching therapeutically-relevant concentrations. These drugs include nucleoside analogs for viral infections, cancer therapeutics, anti-spermatogenic contraceptives, and pro-spermatogenic infertility treatments [11,12,13,14,15,16,17]. Consequently, diseases or disorders may persist if currently available therapeutics cannot effectively bypass the BTB. Several xenobiotics are known to cross the BTB and cause male reproductive toxicity, although the mechanism in which these chemicals cross has been understudied.

BTB penetration of many classes of drugs requires transporter-mediated entry systems; however, studies assessing the expression and localization of these transporters have been limited. Previous studies have reported the contributions and function of the OCTs/OCTNs in the transport of organic cations into polarized monolayers of primary rat SCs plated on Transwell inserts or SCs in suspension [18,19]. The mRNA expression in these cells showed low expression of OCT2 and MATE1, with relatively higher expression levels of OCT1, OCT3, OCTN1, and OCTN2 [18,19]. These studies evaluated the carrier-mediated flux of [^14^C] TEA or [^3^H] carnitine into primary rat SCs. The uptake of [^14^C] TEA was attributed to two transporters (OCT1 and OCTN2), which were referred to as high-affinity transporters in rat SCs [19]. In addition, cellular uptake of [^14^C] TEA revealed a low-affinity component attributed to OCT3 and OCTN1 [19]. However, OCTN2 was the principal mediator of [^3^H] carnitine uptake in primary rat SCs [18], which is an essential chemical for spermatogenesis [20]. Therefore, it was suggested that OCT1 and OCTN2 localized to the basal membrane in rat SCs and OCT3 and OCTN1 localized to the apical membrane. The localization of these transporters may provide a pathway for organic cations and other substrates to bypass the BTB. However, the localization of these transporters in rodent or human tissue and their functional activity in human SCs has not been described. In contrast, the localization of the ENTs has been reported in rat and human testicular tissue [13]. Both rat and human ENT1 are primarily localized to the basal membranes of SCs, whereas ENT2 is localized to the apical membranes. The sequential function of rat ENT1 and ENT2 was confirmed by assessing [^3^H] uridine transport with the selective ENT1-inhibitor, NBMPR, across polarized monolayers of rat SCs plated on Transwell inserts [13,14]. NBMPR inhibits all ENT1 activity at 100 nM; however, high micromolar concentrations are required to block ENT2 activity [21,22,23]. The functional, in vitro data were consistent with basal membrane expression of rat ENT1 and apical expression of ENT2. These two transporters can provide a route for endogenous nucleosides and nucleoside analogs to bypass the BTB.

Transport studies with human SCs assessing the substrate-transporter selectivity and kinetics have been limited due to several factors. One of the prevailing issues is that it is difficult to consistently obtain healthy, adult human testicular tissue to isolate a sufficient number of SCs. Moreover, it has long been thought that SCs cease to proliferate after puberty [24,25,26,27], thus prompting many studies to isolate and use prepubertal rat SCs. However, recent evidence for the existence of proliferative human SCs post-puberty has challenged this idea [28,29,30,31]. Although proliferative human SCs can be isolated, the growth of these primary cells is constrained by the Hayflick limit, which defines the maximum number of divisions that a cell can undergo before entering senescence due to critical telomere shortening [32,33]. Currently, there are several immortalized rat and mouse SC lines including TM4, 15P-1, and SerW3 but the substrate-transporter selectivity or kinetics of rodent transporters may not be representative of data obtained with human orthologs. Therefore, an immortalized human SC line could be useful to study xenobiotic transport across the BTB because of the limitations presented by rodent SCs.

This study characterizes a unique, human telomerase reverse transcriptase-immortalized human SC line (hT-SerC) developed for use in studies that evaluate transporter-substrate selectivity at the BTB. Only one other immortalized human SC line (hS1) has been reported; however, the hS1 cell line is not publicly available and its use in transport studies has not been described [31]. The hT-SerC line described in this study exhibited similar phenotypic characteristics to the primary human SCs from which it was derived and has significantly increased replicative capacity. Moreover, xenobiotic transporter mRNA expression in the hT-SerC line had minimal changes with a few exceptions. This study uses ENT1-mediated transport of the model substrate, uridine, to demonstrate the utility of this human cell model for use in transport studies that directly explore BTB penetration of xenobiotics in humans.

## 2. Materials and Methods

### 2.1. Reagents

[^3^H] Uridine (specific activity: 35.8 or 35.2 Ci/mmol) was purchased from Perkin-Elmer (Waltham, MA, USA). Uridine was purchased from Sigma-Aldrich (St. Louis, MO, USA). The 6-S-[(4-nitrophenyl)methyl]-6-thioinosine (NBMPR) was purchased from Tocris (Bristol, UK). Oligonucleotide primers were designed in house and synthesized by Integrated DNA Technologies (Coralville, IA, USA). Additional reagents were purchased from ThermoFisher Scientific (Waltham, MA, USA) unless otherwise noted.

### 2.2. Cell Culture

Primary human SCs at passage 3 were purchased from MandalMed (San Francisco, CA, USA), propagated as previously described [28], and kept at 35 °C in a humidified 5% CO_2_ incubator with minor differences. Primary cells were grown in DMEM/F12 (Sigma-Aldrich, St. Louis, MO, USA, Catalog #D8900) supplemented with 10 μg/mL human insulin, 2.5 ng/mL EGF, 10% fetal bovine serum, and 1% penicillin and streptomycin. The hT-SerC line was grown and maintained in similar conditions as the primary human SCs with the constant selection pressure of 1 μg/mL puromycin. All cells were washed with Dulbecco’s PBS (DPBS) during routine maintenance. All tissue culture-treated plates and flasks were further coated with 2 μg/cm^2^ poly-l-lysine (Sciencell, Carlsbad, CA, USA, Catalog #0413) before use. Only primary human SCs at passages 4–7 and post-passage 20 hT-SerCs were used in each experiment.

### 2.3. Generation of Stable hTERT-Transduced Human Sertoli Cell Lines

The expression vector, pBABE-puro-hTERT, was a gift from Bob Weinberg (Addgene, Watertown, MA, USA, Catalog #1771) [34]. Following bacterial transformation and culture, the plasmid was subjected to Sanger sequencing to verify the DNA sequence of hTERT using the following primers: pBABE forward (5′-CTTTATCCAGCCCTCAC-3′) and pBABE reverse (5′-ACCCTAACTGACACACATTCC-3′). The pBABE-puro-hTERT plasmid was transiently transfected into the retroviral packaging cell line, Phoenix-AMPHO (ATCC, Manassas, VA, USA, Catalog #CRL-3213), using Lipofectamine 3000 (ThermoFisher Scientific, Waltham, MA, USA, Catalog #L3000001) according to the manufacturer’s protocol and previously described methods [35,36]. Viral supernatant supplemented with 10 μg/mL polybrene from the Phoenix-AMPHO cultures was then harvested and used to infect 60% confluent primary human SCs at passage 4 in a 6-well plate. Selection of antibiotic resistant and stable clones was performed using 1 μg/mL puromycin supplemented media. Clones were continuously cultured under 1 μg/mL puromycin selection pressure at 35 °C in a humidified 5% CO_2_ incubator. Clones that continued to proliferate for more than 20 passages were considered immortal cell lines. Selection of the cell line described in this study involved assessing TERT expression and general growth and morphological characteristics.

### 2.4. RT-qPCR Analysis

Total RNA was isolated from primary human SCs at passages 4–6 and post-passage 20 hT-SerCs using a RNeasy Mini Kit (Qiagen, Hilden, Germany, Catalog #74104) according to the manufacturer’s protocol. RNA quality and concentration were measured using a Spectrophotometer NanoDrop ND-1000 (ThermoFisher Scientific, Waltham, MA, USA). Approximately 1 μg of total RNA was used for first-strand cDNA synthesis with the Cells-to-cDNA II Kit (ThermoFisher Scientific, Waltham, MA, USA, Catalog #AM1722) by following the manufacturer’s protocol using random decamers. Following cDNA synthesis, the reaction mixture was diluted in water before being used for qPCR analysis. qPCR analysis was carried out using PerfeCTa SYBR Green FastMix (Quantabio, Beverly, MA, USA, Catalog #95071-012) and the primers listed in Table 1. Primers were designed using Primer3 software and specificity was validated with an NCBI Nucleotide BLAST search before purchasing from Integrated DNA Technologies (Coralville, IA, USA) and further use according to MIQE guidelines. Non-specific primers for genes were re-designed and tested when necessary. Primer efficiency ranged from 95 to 110% with melting point curves confirming specificity. The PCR reactions were carried out in 96-well plates using a StepOnePlus Real-Time PCR System (Applied Biosystems, Foster City, CA, USA) with the following modified fast 3-step cycling conditions: initial denaturation at 95 °C for 30 s followed by 45 cycles of denaturation at 95 °C for 5 s, annealing at 60 °C for 15 s, and extension for 20 s at 72 °C. Multiple replicates of cDNA for each passage of cells were used. The 2^−ΔΔCt^ method [37] was used to quantify the PCR products for each gene and normalized to *GAPDH*. Negative fold changes represented in the figures were calculated by taking the negative reciprocal value from the 2^−ΔΔCt^ method to signify gene downregulation.

### 2.5. Western Blotting

Total protein was isolated from primary human SCs at passages 4–6 and post-passage 20 hT-SerCs using Triton X-100 lysis buffer (50 mM Tris pH 8.0, 150 mM NaCl, and 1% Triton X-100). The protein concentration was determined using the Pierce BCA Protein Assay Kit (ThermoFisher Scientific, Waltham, MA, USA, Catalog #23225). Protein samples were boiled at 95 °C for 5 min in 4X Laemmli sample buffer (Bio-Rad, Hercules, CA, USA, Catalog #1610747) before gel loading. An equal protein concentration (20 μg) was loaded and resolved on a Novex 4–20% Tris-Glycine mini gel (Invitrogen, Carlsbad, CA, USA, Catalog #XP04205BOX) and transferred onto Trans-Blot Turbo Mini PVDF membranes (Bio-Rad, Hercules, CA, USA, Catalog #1704156). SuperSignal Enhanced Molecular Weight Protein Ladder (ThermoFisher Scientific, Waltham, MA, USA, Catalog #84786) and PageRuler Prestained Protein Ladder (ThermoFisher Scientific, Waltham, MA, USA, Catalog #26616) were used for molecular weight determination and transfer efficiency, respectively. Following protein transfer, membranes were blocked with 5% (wt/vol) non-fat milk in 0.1% Tween 20 in PBS (PBST) or TBS (TBST) for 1 h at room temperature. Membranes were incubated with primary antibodies diluted in the blocking buffer overnight at 4 °C. The primary antibodies used in this study were anti-TERT (1:1000 dilution, Abcam, ab32020), anti-FSHR (1:500 dilution, Abcam, ab75200), and anti-AR (1:500 dilution, Abcam, ab108341) (Abcam, Cambridge, UK).

Following incubation with the primary antibodies, the membranes were washed with PBST or TBST for 10 min at room temperature three times. The membranes were then incubated with a goat anti-rabbit secondary antibody (1:5000 dilution, Invitrogen, Carlsbad, CA, USA, Catalog #62-6520) diluted in blocking buffer for 1 hr at room temperature. After secondary antibody incubation, the membranes were washed with PBST or TBST for 10 min at room temperature three times and incubated with SuperSignal West Femto Maximum Sensitivity Substrate (ThermoFisher Scientific, Waltham, MA, USA, Catalog #34095). Protein bands were visualized using a Bio-Rad ChemiDoc XRS+ imaging system (Bio-Rad Hercules, CA, USA). Following detection, the membranes were partially stripped using Restore™ Western Blot Stripping Buffer (ThermoFisher Scientific, Waltham, MA, USA, Catalog #21059) and washed with PBST or TBST for 20 min. Membranes were re-probed with an anti-beta-actin antibody (1:5000 dilution, Abcam, Catalog #ab227387) (Abcam, Cambridge, UK) and the described protocol was repeated for detection of beta-actin.

### 2.6. Transepithelial Electrical Resistance

Cells were plated at a cell density of 1 × 10^6^ cells/cm^2^ in serum-free media into 24-well Transwell inserts (0.4 μm pore size, 0.33 cm^2^ surface area, Corning, Corning, NY, USA, Catalog #3470) coated with 100 μL of 6.6 μg/mL (2 μg/cm^2^) human fibronectin diluted in DPBS at room temperature. Approximately 200 μL of the cell suspension described above was added into the apical chamber and 800 μL of medium was added into the basal chamber. Cells were left undisturbed at 35 °C in a humidified 5% CO_2_ incubator for 2 days before resistance measurements began. Cells were visually assessed every day after, and measurements were taken every 2 days. Resistance measurements were obtained using a Millicell-ERS Volt-Ohm Meter (MilliporeSigma, Burlington, MA, USA, Catalog #MERS00001) and STX2 chopstick electrodes (World Precision Instruments, Sarasota, FL, USA). Raw measurements were normalized by subtracting the resistance of blank fibronectin-coated inserts without cells from inserts containing cells. Transepithelial electrical resistance (TEER) was calculated by multiplying the normalized resistance measurements by the surface area of the insert.

### 2.7. Uridine Transport Assays

Transport experiments were performed as previously described with minor modifications [38,39,40]. All transport buffers were made with Waymouth’s Buffer (WB; 2.5 mM CaCl·2H_2_O, 28 mM D-glucose, 13 mM HEPES, 135 mM NaCl, 1.2 mM MgCl_2_, 0.8 mM MgSO_4_·7H_2_O, pH 7.4) and 1 μCi/mL [^3^H] uridine (~25 nM) and 5 mM unlabeled uridine, 100 nM NBMPR, or 100 μM NBMPR. Cells were plated into standard 24-well TC plates or Nunc MicroWell 96-well optical bottom plates (ThermoFisher Scientific, Waltham, MA, USA, Catalog #165306) and grown to confluence before each experiment. Cells cultured in 24-well plates were used to assess total [^3^H] uridine uptake after 10 min and cells cultured in 96-well plates were used to assess [^3^H] uridine uptake every minute for 10 min.

Confluent cells plated in 24-well plates were washed once with room temperature WB before incubating with 300 μL WB transport buffer supplemented with 1 μCi/mL [^3^H] uridine (~25 nM) and 5 mM unlabeled uridine, 100 nM NBMPR, or 100 μM NBMPR. Transport was terminated after 10 min by washing the cells three times with WB and the cells were lysed with a lysis solution (0.5 N NaOH, 1% SDS) for 20 min. The NaOH was neutralized using 1 N HCl and the extract was transferred to a 96-well plate. Approximately 200 μL of MicroScint-20 scintillation cocktail (Perkin-Elmer, Waltham, MA, USA, Catalog #6013621) was added to each well with extract before sealing the plate with microplate film and incubating at room temperature for at least 2 hrs before measuring total accumulated radioactivity measured using a Wallac 1450 MicroBeta TriLux liquid scintillation counter (Perkin-Elmer, Waltham, MA, USA).

Confluent cells plated in 96-well plates were washed once with room temperature WB before incubating one column of wells with 50 μL WB transport buffer supplemented with 1 μCi/mL [^3^H] uridine (~25 nM) and 5 mM unlabeled uridine, 100 nM NBMPR, or 100 μM NBMPR every minute from 0 to 10 min. Transport was terminated after the first column of wells had been incubated for 10 min by washing the cells twice with WB using a Biotek 405 LS Microplate Washer (BioTek, Winooski, VT, USA). After washing, 200 μL of MicroScint-20 scintillation cocktail was added to each well and the steps described above were followed to quantify uptake.

To determine the IC_50_ for NBMPR, cells were plated into Nunc MicroWell 96-well optical bottom plates (ThermoFisher Scientific, Waltham, MA, USA, Catalog #165306) and grown to confluence before each experiment. Cells were washed once with room temperature WB using the Biotek plate washer before beginning the experiment. Transport was initiated by adding 50 μL of transport buffer containing 1 μCi/mL [^3^H] uridine (~25 nM) and serially diluted NBMPR (0–10 nM) to the cells. Transport was terminated after 10 min by washing the cells twice with WB using the Biotek plate washer. After washing, total accumulated [^3^H] uridine was measured as described above. Non-transport mediated uptake of [^3^H] uridine was subtracted from each data point to separate transport-mediated uptake for data analysis. Data were normalized as a percentage of the uninhibited [^3^H] uridine uptake control.

### 2.8. Affymetrix GeneChip Microarray Analysis

Total RNA was isolated from primary human SCs at passages 4–6 and post-passage 20 hT-SerCs using a RNeasy Mini Kit (Qiagen, Catalog #74104) according to the manufacturer’s protocol. RNA quality and concentration were measured using a Spectrophotometer NanoDrop ND-1000 (ThermoFisher Scientific, Waltham, MA, USA). Approximately 100 ng of total RNA in 10 μL for both cells was submitted to the University of Arizona Cancer Center Genomics Shared Service Core to perform whole genome RNA expression analysis on an Affymetrix GeneChip human Clariom D microarray (ThermoFisher Scientific, Waltham, MA, USA, Catalog #902922). Gene expression signals were collected as log_2_ averages for each cell and the fold change was calculated relative to the primary human SCs. The microarray data were deposited in the NIH NCBI GEO database with the series accession number: GSE158400.

### 2.9. Statistical Analysis

Each experiment was completed with cells cultured from at least three separate passages with multiple replicates. Western blots are representative of at least three independent experiments. Generation of IC_50_ values and statistics were completed using GraphPad Prism 8 (GraphPad Software, San Diego, CA, USA). The IC_50_ values for NBMPR inhibition of [^3^H] uridine uptake were reported with 95% confidence intervals. Data are reported as the mean ± S.D. unless otherwise noted. Statistical analysis was not performed for the RT-qPCR data since fold-change values are not normally distributed.

## 3. Results

### 3.1. Immortalization of Human Sertoli Cells with Human Telomerase Reverse Transcriptase

**Figure 1:**
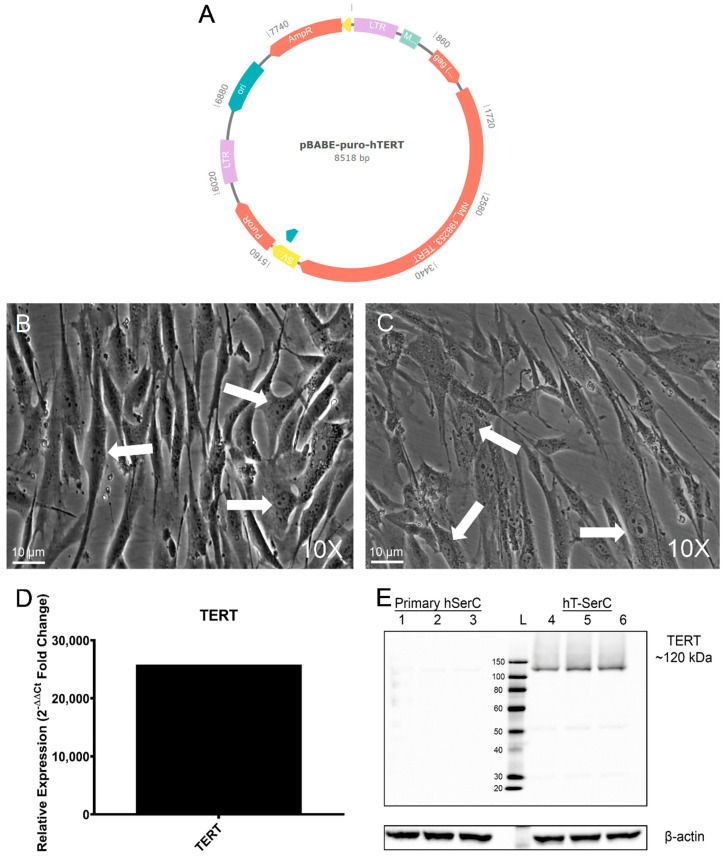
(**A**) Expression vector for pBABE-puro-hTERT [34] used to transfect Phoenix-AMPHO packaging cells for subsequent transduction into primary human SCs. Phase contrast microscopy images at 10× magnification of (**B**) primary human SCs at passage 4 and (**C**) immortalized hT-SerCs at passage 21. White arrows indicate tripartite nucleoli [41] within the nucleus of each cell. (**D**) Relative mRNA expression of human *TERT* assessed by RT-qPCR using the 2^−ΔΔCt^ method. Ct values were normalized to *GAPDH* as the reference gene. (**E**) Representative Western blot of three independent experiments for the expression of human TERT in primary human SCs at passages 4 and 6 (lanes 1–3) and post-passage 20 hT-SerCs (lanes 4–6). The molecular weight ladder is denoted as L. Beta-actin served as the loading control.

Primary human SCs obtained from MandalMed were previously isolated from a 36-year-old donor and characterized by their morphology, gene expression profiles, and function [28,29]. The primary human SCs were noted for their proliferative capacity; however, these cells can only be passaged a limited number of times before reaching the Hayflick limit and entering cellular senescence [32,33]. Therefore, an immortalized human SC line with unlimited proliferative capacity, typical characteristics of SCs, and similar xenobiotic transporter expression profiles would be useful for stable and high-throughput in vitro drug transport studies. Proliferative primary human SCs at passage 4 were transduced with a retrovirus encoding hTERT and puromycin resistance produced by a vector-transfected Phoenix-AMPHO packaging cell line (Figure 1A). Stable hTERT-expressing clones were selected and expanded under 1 μg/mL puromycin selection pressure for over 20 passages before being considered immortal, hereinafter referred to as hTERT human Sertoli cells (hT-SerCs). In contrast, the primary human SCs underwent complete senescence after 12 passages. hT-SerCs could be passaged every 4 to 5 days by splitting a nearly confluent T-75 flask (~1–2 million cells) at 1:3 to 1:5. Experiments performed with primary human SCs were used between passages 4 and 7 and hT-SerCs were used only after passage 20.

Both primary human SCs and hT-SerCs generally assumed a large, fibroblastic morphology with tripartite nucleoli [41] (Figure 1B,C). RT-qPCR analysis of human *TERT* expression in hT-SerCs indicated a fold change in expression relative to primary human SCs of over 25,800 (Figure 1D). Similarly, microarray analysis showed an approximately 10,000-fold increase in expression (Figure S1), indicating successful transduction and expression of hTERT in these cells. Western blot analysis confirmed that the protein expression of human TERT in hT-SerCs was greatly increased compared to the primary human SCs (Figure 1E). Light bands at 120 kDa were noted in the primary human SC protein samples and were attributed to normal, residual TERT expression as observed in other normal, somatic cells.

### 3.2. Transepithelial Electrical Resistance

**Figure 2:**
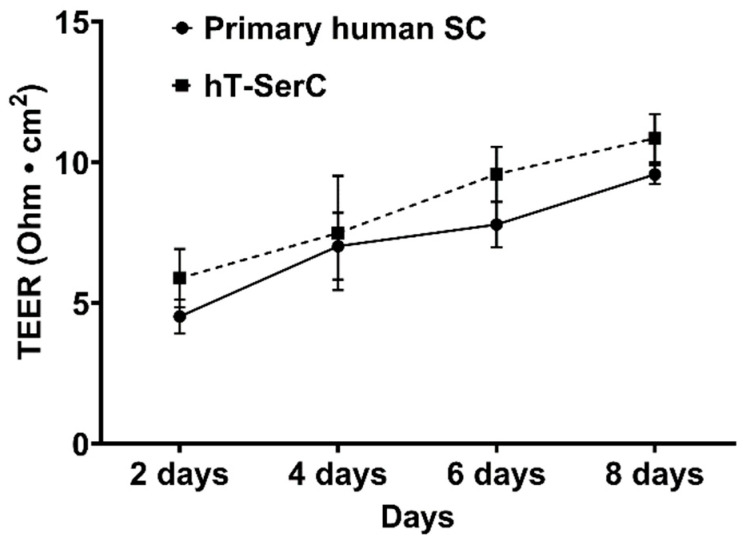
Transepithelial electrical resistance of primary human SCs at passages 4–6 and post-passage 20 hT-SerCs plated on 24-well Transwell inserts (0.4 μm pore size, 0.33 cm^2^ surface area) coated with 100 μL of 6.6 μg/mL (2 μg/cm^2^) human fibronectin. Resistance values were measured every two days for eight days and net resistance values for each insert were calculated by subtracting from cell-free coated inserts. TEER was calculated by multiplying the normalized resistance measurements with the surface area of the insert. Data are represented as the mean ± S.D. of three independent experiments.

The formation of an in vitro BTB was evaluated by plating primary human SCs at passages 4–6 or post-passage 20 hT-SerCs cells on semi-permeable 24-well Transwell inserts coated with fibronectin. TEER was used to measure cell monolayer permeability and cells were assessed visually each day and TEER measurements taken every other day. TEER values gradually increased over 8 days for both cells; however, cell monolayers contracted from the edges of the insert beyond 8 days. As a result, the TEER measurements for contracted monolayers decreased drastically and were unrecoverable. Both primary human SCs and hT-SerCs reached a peak of ~10 Ohms·cm^2^ after 8 days (Figure 2).

### 3.3. Gene Expression Profiles for Typical Sertoli Cell Markers

**Figure 3:**
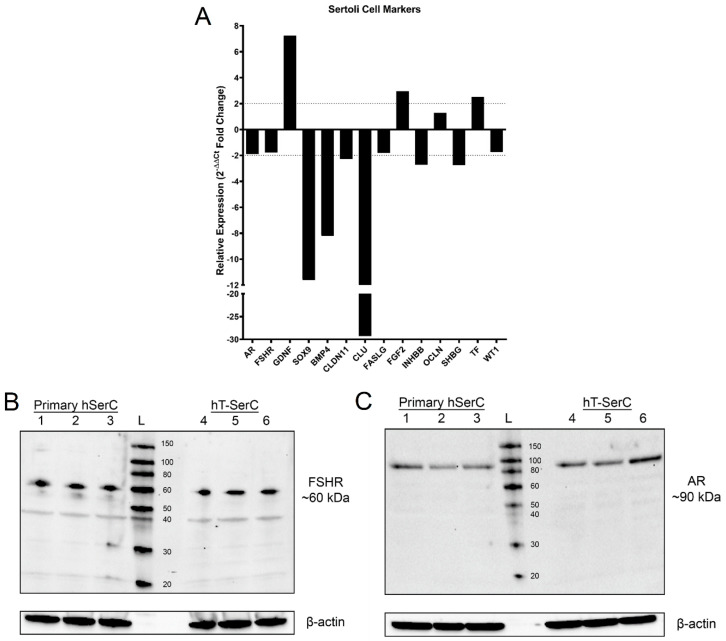
(**A**) Relative mRNA expression of various human SC marker genes assessed by RT-qPCR using the 2^−ΔΔCt^ method. Ct values were normalized to *GAPDH* as the reference gene. (**B**) Representative Western blot of three independent experiments for the expression of human FSHR in primary human SCs at passages 4–6 (lanes 1–3) and post-passage 20 hT-SerCs (lanes 4–6). (**C**) Representative Western blot of three independent experiments for the expression of human AR in primary human SCs at passages 4–6 (lanes 1–3) and post-passage 20 hT-SerCs (lanes 4–6). The molecular weight ladder is denoted as L. Beta-actin served as the loading control.

**Figure 4:**
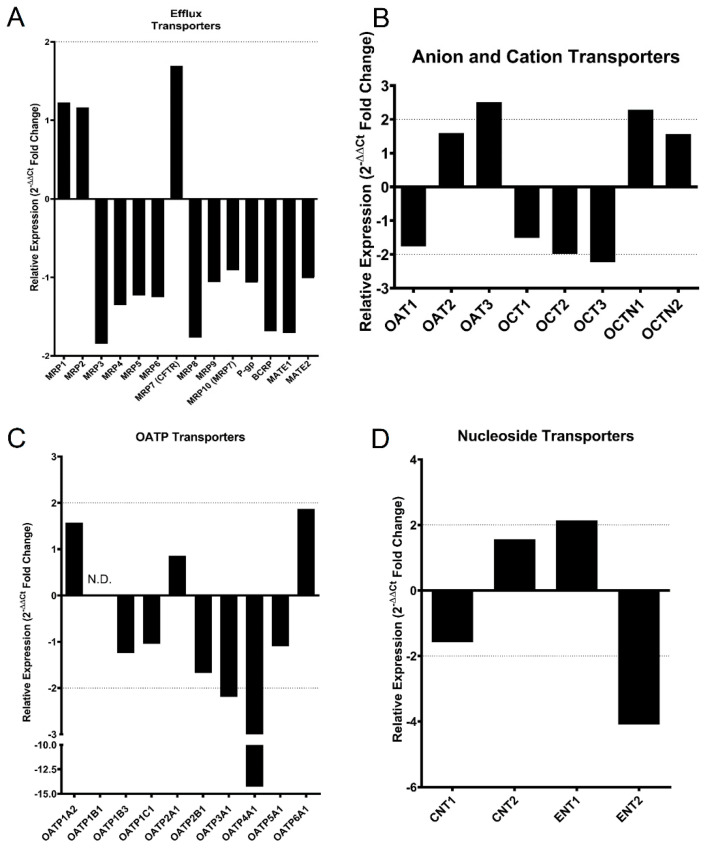
(**A**) Relative mRNA expression of various human xenobiotic efflux transporter genes assessed by RT-qPCR. (**B**) Relative mRNA expression of human anion and cation transporter genes in the SLC22A family. (**C**) Relative mRNA expression of human organic anion transporting polypeptide transporter genes in the SLC21 family. *OATP1B1* expression was not detected in either cell type and denoted as N.D. (**D**) Relative mRNA expression of human concentrative and equilibrative nucleoside transporters. All fold-change values were calculated using the 2^−ΔΔCt^ method. Ct values were normalized to *GAPDH* as the reference gene.

SCs express a number of genes that are typically used as cell-specific markers to distinguish between the cells of the testes. RT-qPCR analysis of human *AR*, *FSHR*, *GDNF*, *SOX9*, *BMP4*, *CLDN11*, *FGF2, OCLN,* clusterin (*CLU*), Fas ligand (*FASLG*), inhibin subunit beta b (*INHBB*), sex hormone-binding globulin (*SHBG*), transferrin (*TF*), and Wilms’ tumor protein (*WT1*) revealed minor changes to most SC markers in post-passage 20 hT-SerCs relative to primary human SCs at passages 4–6. A 2-fold difference threshold in expression was chosen to identify more robust changes in RT-qPCR gene expression as indicated by the dashed lines (Figure 3 and Figure 4). For microarray analysis, a 3-fold difference threshold was chosen as recommended by correspondence with Dr. George Watts (Figures S1–S3). Fold-change differences around 1 or −1 for both assays indicate minimal change in expression between cells due to the reported values being relative to primary human SCs. *GDNF* had a 7.3-fold increase in mRNA expression relative to primary human SCs, which correlated with an 18.1-fold increase in expression as determined by microarray analysis (Figure 3A and Figure S2). A minor 2.9-fold and 3.2-fold increase in *FGF2* expression was also observed in RT-qPCR and microarray analysis, respectively (Figure 3A and Figure S2).

In contrast, SC markers with a decrease in mRNA expression indicate a divergence from the normal SC phenotype. RT-qPCR analysis for *SOX9*, *BMP4*, and *CLU* expression in hT-SerCs was found to be approximately 11.6-fold, 8.2-fold, and 29.3-fold lower compared to primary human SCs, respectively (Figure 3A). Correspondingly, a 5.5-fold, 45.5-fold, and 109.0-fold decrease in expression for *SOX9*, *BMP4*, and *CLU* was detected by microarray analysis (Figure S2). *CLDN11*, *INHBB*, *SHBG*, and *TF* crossed the RT-qPCR 2-fold change threshold in either direction but had negligible differences in microarray analysis. These results suggest that hT-SerCs maintain notable phenotypic similarities with primary human SCs, although there are detectable differences.

Due to the roles of follicle-stimulating hormone and androgens in regulating crucial processes in SC regulation of germ cell development and managing the hypothalamic-pituitary-gonadal axis, the protein expression of the corresponding receptors (androgen receptor [AR] and follicle-stimulating hormone receptor [FSHR]) was evaluated. Western blot analysis indicated a negligible difference in FSHR expression between both cells (Figure 3B), which was expected due to minor differences in mRNA expression (Figure 3A and Figure S2). Additional lighter bands for FSHR at 20, 30, and 40 kDa were noted and attributed to splice variants of the protein [42,43,44]. Similarly, there were inconsequential differences in protein expression of AR, supported by minimal changes to mRNA expression (Figure 3A,C and Figure S2).

### 3.4. mRNA Expression of Common Xenobiotic Transporters

Although there are some differences in SC marker expression between both cells, the hT-SerCs were generated to study the xenobiotic transporters found at the BTB. Uptake and efflux transporters found at the BTB regulate the flux of chemicals into and across SCs. Many efflux transporters have been identified at the BTB [45,46,47,48,49,50,51,52]; however, there are many pharmacologically-relevant uptake transporters in SCs that are understudied. This prompted further analysis of mRNA expression for common xenobiotic transporters in both primary human SCs at passages 4–6 and post-passage 20 hT-SerCs. RT-qPCR analysis of common efflux transporters revealed negligible differences (Figure 4A). Similarly, microarray analysis for the same efflux transporters did not show differences past the fold-change threshold (Figure S3A). The decrease in *BCRP* expression by microarray analysis was close to the threshold but was not considered a robust change (Figure S3A).

Typical anion and cation transporters that transport xenobiotics include the SLC21 (SLCO/OATP) and the SLC22 families, which have varying organ-specific expression levels. Therefore, the mRNA expression of several members of these two families was evaluated. *OAT3*, *OCT3*, and *OCTN1* expression had crossed the fold-change threshold (Figure 4B); however, the microarray only confirmed an increase in expression for *OCTN1* and *OCTN2* (Figure S3B). In RT-qPCR and the microarray, *OCTN1* was upregulated by 2.3-fold and 3.5-fold, respectively. The expression of two OATP transporters had changes determined to be robust by RT-qPCR analysis (Figure 4C). The mRNA expression of *OATP3A1* and *OATP4A1* was observed to be 2.2-fold and 14.3-fold downregulated in hT-SerCs. *OATP4A1* downregulation was consistently observed by RT-qPCR in three independent experiments. However, microarray analysis for the same OATP transporters revealed no significant change in mRNA expression past the threshold. *OATP1B1* mRNA was not detected in either primary human SCs or hT-SerCs in three independent RT-qPCR experiments, although *OATP1B1* mRNA transcripts were detectable in the microarray (Figure S3C).

Endogenous nucleosides are important for RNA and DNA synthesis in cells, which are typically transported by the CNTs or ENTs. Protein expression of the CNTs in SCs is limited [13], thus requiring the ENTs to mediate the majority of nucleoside transport into SCs. The expression of ENT1 and ENT2 in SCs has been reported with extensive kinetic parameters being evaluated [13,14,38]. Therefore, the mRNA expression of these transporters was evaluated in the primary human SCs and hT-SerCs. *CNT1* and *CNT2* mRNA was detectable in both cells; however, the expression change relative to primary human SCs was inconsequential (Figure 4D and Figure S3D). The expression of *ENT1* was upregulated by 2.1-fold and 3.8-fold by RT-qPCR and microarray analysis, whereas *ENT2* was downregulated by 4.1-fold as shown by RT-qPCR but not in the microarray (Figure 4D and Figure S3D).

### 3.5. Inhibition of [^3^H] Uridine Uptake by NBMPR

**Figure 5:**
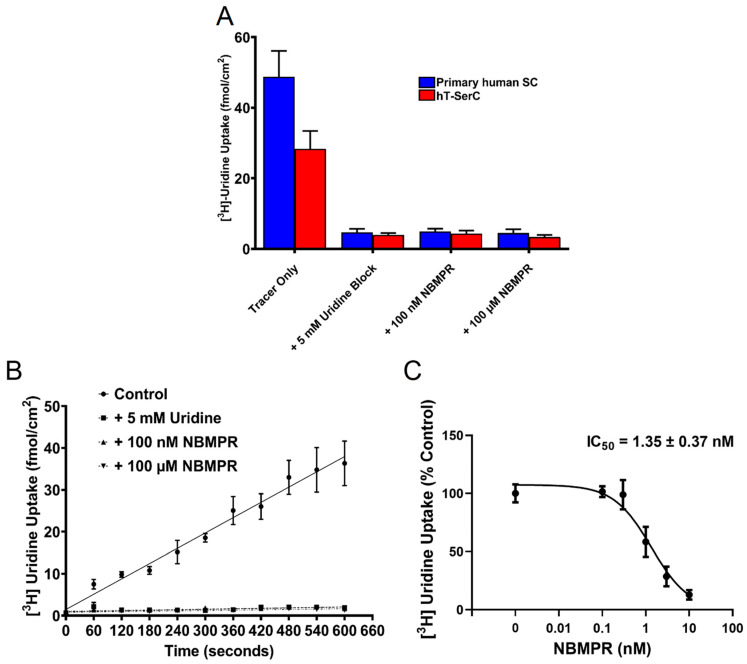
(**A**) Total [^3^H] uridine accumulation into primary human SCs at passages 4–7 and post-passage 20 hT-SerCs after 10 min of uptake. Approximately 85–90% of uptake is blocked by 5 mM unlabeled uridine, 100 nM NBMPR, and 100 μM NBMPR. Data are represented as the mean ± S.D. of five independent experiments. (**B**) Time-course experiment of [^3^H] uridine uptake measured every minute for 10 min. [^3^H] Uridine uptake is effectively linear over 10 min and approximately 85–90% of uptake is blocked by unlabeled uridine or NBMPR. The best-fit line represents a one-phase association. Data are represented as the mean ± S.E.M. of three independent experiments. (**C**) Inhibition of [^3^H] uridine uptake by serially increasing concentrations of NBMPR from 0 to 10 nM. Transport was terminated after 10 min. The best-fit line was fitted using the monophasic inhibition model described by Equation (1). Data are represented as the mean ± S.E.M. of three independent experiments.

The expression of the ENTs in SCs has been well described and provides a route for endogenous nucleosides and nucleoside analog drugs to cross the BTB [13,14]. To validate the presence and function of the ENTs in hT-SerCs, the cellular uptake of a model substrate, uridine [13,14,22,38,53,54,55,56], was measured by transport assays. Total accumulation of ~25 nM [^3^H] uridine into primary human SCs at passages 4–7 and post-passage 20 hT-SerCs after 10 min was measured in the presence and absence of unlabeled uridine or two concentrations of NBMPR. NBMPR is a selective ENT1 inhibitor that effectively blocks all ENT1 activity at 100 nM; however, it also blocks all ENT2 activity at a higher concentration of 100 μM. Approximately 85–90% of [^3^H] uridine uptake in the primary human SCs and hT-SerCs was reduced in the presence of unlabeled uridine or both concentrations of NBMPR (Figure 5A). The total accumulation of [^3^H] uridine in primary human SCs after 10 min was approximately ~50 fmol/cm^2^ (Figure 5A) Interestingly, the maximum uptake of [^3^H] uridine was ~30–40% lower in hT-SerCs (~30 fmol/cm^2^); however, unlabeled uridine and NBMPR continued to block uptake to the same extent. These data suggest that uridine uptake into both cells is primarily mediated by ENT1 with minimal contribution by ENT2.

Therefore, subsequent transport experiments focused on the characteristics of ENT1-mediated transport in hT-SerCs. Accumulation of ~25 nM [^3^H] uridine was measured over a period of 10 min with data points collected at each minute. The uptake of [^3^H] uridine was linear during this time and was also reduced by 85–90% in the presence of 5 mM unlabeled uridine and either 100 nM or 100 μM NBMPR (Figure 5B). These data indicate the predominant functional expression of ENT1 at the plasma membrane due to the lack of an additional ENT2-mediated component being blocked by 100 μM NBMPR over the time period.

A dose–response curve generated by using serially diluted concentrations of NBMPR from 0 to 10 μM inhibited [^3^H] uridine uptake with a monophasic pattern (Figure 5C). This is consistent with the dominant expression of ENT1 in these cells. The apparent IC_50_ of NBMPR on ENT1-mediated [^3^H] uridine uptake was determined using a modified Equation (1) [38,57]:

(1) $$J=\frac{J\mathrm{app}-\max\;\times \;\left[T\right]}{{\mathrm{IC}}_{50}\;+\;\left[S\right]}$$

J represents total uridine transport, J_app-max_ is the apparent J_max_ for uridine times the ratio of IC_50_ to the K_m_ of for uridine transport (i.e., J_app_ = J_max_(IC_50_/K_m_); (fmol/cm^2^·min)), [T] is the [^3^H] uridine concentration, and [S] is the NBMPR concentration. Results were normalized to percent of the control. The apparent IC_50_ value of NBMPR on ENT1-mediated [^3^H] uridine uptake is 1.35 ± 0.37 nM (95% CI: 0.57, 2.12). The R^2^ for the goodness of fit of Equation (1) was 0.86.

## 4. Discussion

Primary SCs are preferred when studying the complex transport profiles at the BTB [13,14,18,19,58], because of the likelihood that they closely approximate in vivo transport. However, the isolation and maintenance of primary human SCs for studying transport at the BTB is often tedious and requires a consistent supply of healthy testicular tissue. Moreover, primary cells have limited proliferative capacity due to the Hayflick limit and can express genetic changes after several passages. Interestingly, a previous study observed no statistical differences to *ENT* or *CNT* mRNA expression between freshly isolated rat SCs and rat SCs cultured for 6 days [14], suggesting gene expression may be relatively stable for a period of time post-isolation. In any case, this precludes the use of primary human SCs, since obtaining large numbers of healthy human testes for SC isolation and further studies is notably problematic and unrealistic. Consequently, primary rat SCs are often isolated from pre-pubertal rats, but this involves the sacrifice of many animals to sustain xenobiotic transport studies on a regular basis. There are commercially-available immortalized rodent SCs and xenobiotic transport using immortalized mouse SCs (TM4 cells) has been described [50,56,59]. However, species differences between rodent and human transporters for both substrate-transporter selectivity and kinetics, or the lack of relevant orthologs [60], dictate the need for an immortalized human SC line to study the transport dynamics at the human BTB. Interestingly, a previous study observed MRP4 localization at the apical membrane of SCs in normal rodent seminiferous tubules in contrast to basal membrane localization in humans and rhesus macaques [46]. A separate study observed similar apical membrane localization of MRP4 in rats upon neonatal treatment with zearalenone, although staining in normal rat testes revealed basal membrane localization [47]. These conflicting results introduce a unique issue when using rodent models to study transport across the BTB, because those models may not be completely representative of BTB transport in humans. Although genetic changes tend to occur after cell isolation and subsequent culturing from the original tissue source, gene expression in primary human SCs are the closest approximation to the in vivo environment in human testes. Due to the limitations of obtaining enough freshly isolated human SCs for large-scale transport studies, a convenient and high-throughput model that closely approximates in vivo conditions or primary human SCs is necessary to support the discovery and development process for drugs that target the male reproductive tract. The present study describes the first immortalized human SC line specifically developed to study xenobiotic transporters at the human BTB to the closest approximation in contrast to modeling true SC physiology.

The hT-SerCs described in this study were shown to stably overexpress TERT at the transcriptional and translational level compared to the minor expression in primary human SCs. In addition, this cell line retained similar morphological characteristics to the primary human SCs from which they were derived, and cells described in previous studies (Figure 1B,C) [28,29,30,31]. The primary human SCs have been well characterized [28,29], thus presenting a convenient baseline to compare the phenotype of the hT-SerCs. Both primary human SCs and hT-SerCs displayed fibroblastic-like morphology with distinct tripartite nucleoli, which are characteristic of SCs [41]. The formation of tight junctions in these cells plated on Transwell inserts was assessed by measuring transepithelial electrical resistance. Peak measurements were limited to ~10 Ohms·cm^2^ (Figure 2), which is consistent with values obtained in previous studies [28] (when correctly calculated as Ohms·cm^2^ [61]). After 8 days in culture, the SCs began to contract from the edges of the Transwell inserts and resistance measurements drastically decreased with no observable recovery afterwards. Due to the low TEER measurements observed in primary human SCs and hT-SerCs, further assessment of tight junction protein expression was not warranted. Although tight junction proteins may be expressed by each cell, the typical strong cell–cell interactions between these proteins was not observed with TEER measurements. While the previous study observed polarized secretion of galectin-1, the comparatively low TEER values suggest that the barrier function of these SCs in culture is not representative of the in vivo BTB. The electrical resistance of blood–organ barriers has been estimated in vivo [61]; however, determining a true TEER value for these epithelial barriers is impractical in whole animals. MDCK and Caco-2 cells are classical models of epithelial cells forming tight junctions and are largely impermeable to paracellular flux of organic solutes. The TEER values observed in these cell lines range from 200 to 300 Ohms·cm^2^ in MDCK cells and exceed 1000 Ohms·cm^2^ in Caco-2 cells [61,62]. These values are a stark contrast to those obtained with primary human SCs or hT-SerCs, indicating that human SCs may not be appropriate to study transepithelial transport. It is possible that the unique morphological features of SCs in culture preclude the formation of a tight barrier observed in MDCK or Caco-2 cells. MDCK and Caco-2 cells adopt a distinctly round, cobblestone-like morphology that may promote close cell–cell interactions. On the other hand, the SCs described in this study and in other studies adopt a large, fibroblastic-like morphology that may not be as suitable for forming strong cell–cell interactions in culture. SCs in testicular tissue are noted for their irregular shape with many indentations to accommodate large, developing germ cell nuclei and plasma membranes. Because the SCs are effectively sitting flat on Transwell inserts, they may not be able to elongate “vertically” as they normally would. As a result, tight junctions may not be able to effectively form between SCs regardless of their expression and limits the use of these cells when studying the transepithelial transport of xenobiotics despite the use of various cell culture additives or extracellular matrices. A more complex three-dimensional culture model with or without supplementary cells such as peritubular myoid cells or germ cells may be required to establish a functional, polarized epithelia, although further studies are required. However, these cells are still valuable for studying xenobiotic uptake or efflux transport independently on standard tissue culture-treated plates as demonstrated in this study.

The mRNA expression by RT-qPCR analysis of several typical SC markers (*GDNF*, *SOX9*, *BMP4*, *CLU*, and *FGF2*) in these cells diverged from primary human SCs; however, many other genes had nearly negligible differences as evidenced by microarray analysis for the same genes. GDNF and FGF2 are secreted growth factors that promote spermatogonial stem cell self-renewal [63,64], and the increased expression of these genes may be useful for studying interactions with germ cell replication and development. However, additional studies are required to identify the importance of *GDNF* and *FGF2* overexpression in hT-SerCs. In contrast, the downregulation of *SOX9*, *BMP4*, and *CLU* may correspond with the increased proliferative nature of hT-SerCs. SOX9 is a transcription factor that is primarily responsible for sex determination and is upregulated in mature, differentiated SCs; however, it is decreased in immature, proliferative SCs [65]. Therefore, it was expected that *SOX9* mRNA expression decreased by 11.6-fold (Figure 3A) in the proliferative hT-SerCs. This decrease in expression of this transcription factor and the promotion of a more immature phenotype may correlate with the inability of hT-SerCs to form a tight, polarized epithelia on Transwell inserts. However, this does not explain the inability for primary human SCs to form a tight, polarized epithelia. With respect to other SC markers, BMP4 regulates spermatogonial stem cell self-renewal [66] and clusterin regulates germ cell apoptosis and clearance of cellular debris [67]. However, the lack of germ cells in these SC monocultures may explain *BMP4* and *CLU* mRNA downregulation because the synthesis of the corresponding proteins is no longer required. Additionally, BMP4 is known to inhibit GDNF expression and may explain the opposite trends in mRNA expression. These two genes are unlikely to significantly affect tight junction formation and do not explain the low TEER observations found in primary human SCs. Several genetic and physical factors may affect the inability for primary human SCs to form a tight barrier in the Transwell insert culture model; however, further studies will be necessary to investigate this phenomenon. Despite these larger changes in SC marker gene expression, the expression of other genes remained within the 2-fold change threshold. Notably, the mRNA and protein expression of AR and FSHR remained unchanged after immortalization and over 20 additional passages. AR and FSHR are fundamental receptors expressed on SC membranes and the similar expression of these receptors in hT-SerCs may be useful in future studies. Despite the similarities and differences in gene expression of SC markers, these hT-SerCs were developed to study the role and function of xenobiotic transporters at the BTB. This immortalized cell line may be useful in studying other aspects of SC biology; however, there are differences that must be noted.

Xenobiotic transporter mRNA expression varied within each group of transporters, but the expression of a majority of uptake and efflux transporters remained the same or had minimal changes. By RT-qPCR analysis, the significant changes occurred with SLC22A anion and cation transporters (*OAT3*, *OCT3*, and *OCTN1*), OATP transporters (*OATP3A1* and *OATP4A1*), and *ENT2* (Figure 4B–D). Changes to these transporters was classified as ‘robust’ (>2-fold change), although many of these changes were unsupported by microarray analysis. These larger changes detected by RT-qPCR analysis may be due to already minimal mRNA expression in primary human SCs with small increases or decreases in hT-SerCs amplifying fold-change values after analysis with the 2^−ΔΔCt^ method. It is important to note that not all of the xenobiotic transporters discussed in this study are expected to have high expression in SCs. Xenobiotic transporter expression levels have been reported in rat SCs [68]; however, these may differ greatly when compared to transporter expression in human SCs. Additionally, there are potential expression and functional differences attributed to interindividual variability. Inter-individual variability may be one factor in explaining the minimal expression of some xenobiotic transporters versus others in the primary human SCs and hT-SerCs described in this study. Nevertheless, the localization and function of highly expressed transporters is essential for the penetration of xenobiotics across the BTB. While the use of gene expression systems to produce xenobiotic transporter-overexpressing cell lines has been common for studying individual substrate-transporter kinetics, a single cell line that endogenously expresses a collection of transporters is preferable when studying which transporters are involved in the flux of xenobiotics with unknown trafficking processes. Many xenobiotics are known to cross the BTB and elicit therapeutic or toxic effects [11,12,13,14,15,16,17], although the BTB penetration mechanism for a sizeable portion of these compounds is unknown. The hT-SerCs described in this study express several uptake transporters; however, they also express efflux transporters that contribute to the net disposition of many xenobiotics. The function of the BTB describes both the tight junctions that form between SCs and the function of efflux transporters that limit drug accumulation in SCs. Compounds that may be effectively transported into renal cells, hepatocytes, or other cell types may not accumulate in SCs due to the expression of efflux transporters such as P-gp, BCRP, or the MRPs contributing to BTB function [45,46,47,48,49,50,51,52]. Unlike single transporter-expressing cell lines, the collective influence of these uptake and efflux transporters expressed in hT-SerCs can help delineate which compounds effectively cross into and accumulate within SCs. Furthermore, the use of selective and broad-spectrum transporter substrates or inhibitors in combination with the compound of interest can help identify the transporters responsible for mediating flux across SCs. The preliminary screening for the transport and accumulation of these compounds in hT-SerCs will save many resources compared to screening compounds in multiple cell lines to achieve the same result. The hT-SerCs reported here can be used as an important tool for identifying xenobiotics that use carrier-mediated pathways to cross the SC epithelium.

Previous studies identified the ENTs as the primary mediators of endogenous nucleoside transport in rodent SCs [13,14], with evidence of their polarized expression in human SCs [13,14,69]. Unlike many other cell models, such as primary rat SCs, TM4 cells, or HeLa S3 cells that express both ENT1 and ENT2 at the plasma membrane, the primary human SCs and hT-SerCs described in this study predominantly express ENT1. However, *ENT2* mRNA transcripts were observed in both RT-qPCR and microarray analysis of primary human SCs and hT-SerCs. It is worth noting that ENT2 can be expressed at the nuclear membrane to regulate endogenous nucleoside transport into the nucleus for DNA synthesis [70], although the role of the decrease in *ENT2* mRNA expression in hT-SerCs requires further investigation. The contribution of ENT2-mediated transport at the nuclear membrane would not be identified by the functional analysis used in this study, because total intracellular [^3^H] uridine accumulation is measured based on transport across the plasma membrane. These differences in plasma membrane expression may be attributed to species differences, inter-individual variability, or tissue-specific expression of each transporter. More importantly, the uptake of [^3^H] uridine into primary human SCs and hT-SerCs was linear over 10 min. Although the maximum capacity for [^3^H] uridine uptake was 4- to 5-fold lower compared to HeLa S3 cells after 7 min, the linear uptake and response to NBMPR inhibition was consistent with previous studies [38]. In addition, the apparent IC_50_ value of 1.35 ± 0.37 nM for NBMPR of ENT1 was well within the range of low nanomolar values reported in previous studies using a variety of cell models [22,38,53,54,55,56]. These data suggest that ENT1 kinetics for nucleoside analogs and other exogenous substrates can be adequately modeled with hT-SerCs with the intent to evaluate xenobiotic transport across the BTB.

In conclusion, hT-SerCs have a significantly enhanced lifespan while retaining many phenotypic characteristics of the original primary human SCs from which they were derived. The phenotypic characteristics of these cells also appears to be stable, with no apparent morphological or growth changes being detected after 40 passages. Consequently, hT-SerCs may be suitable for other applications that involve SC biology, but further studies are necessary to explore their potential. The xenobiotic transporter expression in hT-SerCs appears to have minimal changes at the transcriptional level, with some exceptions. Importantly, the overall transport of [^3^H] uridine into these cells remains unchanged even after hundreds of population doublings. These data suggest that hT-SerCs may be a robust and suitable model for studying transport of selected xenobiotics at the BTB. As a result, these cells may be a valuable tool for identifying the transport mechanisms of known testicular toxicants, antivirals, cancer chemotherapeutics, chemical contraceptives, and infertility treatments across the BTB. The identification of these pathways will be essential for the development of novel therapeutics that require access to the MGT.

## Figures and Tables

**Table 1:** List of forward and reverse RT-qPCR primers used in this study.

Gene Name	Forward Primer	Reverse Primer
*GAPDH*	5′-CGACCACTTTGTCAAGCTCA-3′	5′-CCCTGTTGCTGTAGCCAAAT-3′
*TERT*	5′-CGGTGTGCACCAACATTAC-3′	5′-GGGTTCTTCCAAACTTGCTG-3′
*AR*	5′-CCATAAGCCACTTGGATGCT-3′	5′-TGTCATGTCTGAGGCACTCC-3′
*FSHR*	5′-AAATGGAGCTTGCATTCTGG-3′	5′-TCCTTCCCAGATTCTCCTGA-‘3
*GDNF*	5′-TCTTCATGGTTCTGCCCTTC-3′	5′-GCTGGGGTTTGTCACTGTTT-3′
*SOX9*	5′-AGACCTTTGGGCTGCCTTAT-3′	5′-TAGCCTCCCTCACTCCAAGA-3′
*BMP4*	5′-ACTGGCTGACCACCTCACT-3′	5′-GTTCAGTGGGCACACAACAG-3′
*CLDN11*	5′-TTCACGGTATTGCAGTGGTAA-3′	5′-GTTTCTGATTGCTGCCCATT-3′
*CLU*	5′-GCTGCAAATGGAAGCTTTTC-3′	5′-TTCTGGGCACCAAATGTTTT-3′
*FASLG*	5′-CCATGTGAAGAGGGAGAAGC-3′	5′-AAGACAGTCCCCCTTGAGGT-3′
*FGF2*	5′-CCATCCTTTCTCCCTCGTTT-3′	5′-TTCCCTCCAATGTTTCATTCA-3′
*INHBB*	5′-TGAACGCACATGACATAGCA-3′	5′-ACGTGGCACTTGGACATCTA-3′
*OCLN*	5′-ATGCCTAGCTACCCCATCT-3′	5′-AATGCCAATCCTGCATTCTC-3′
*SHBG*	5′-CTCCCCTCCTTAACCTCTGG-3′	5′-AGAGGTTTCCTTCCCCTCAA-3′
*TF*	5′-AAGCCTGCACTTTCCGTAGA-3′	5′-AAGCCTGCACTTTCCGTAGA-3′
*WT1*	5′-TACCTCCTTGCACAAATGGA-3′	5′-CCTGGACCATCCCCTATTTT-3′
*MRP1*	5′-CTCGTTAGAGCCCAAAGTGG-3′	5′-ACAAAAGGATCCCCCAAAAC-3′
*MRP2*	5′-CCGTATCAGGTTTGCCAGTT-3′	5′-TGGAGGTGATCCAGGAAAAG-3′
*MRP3*	5′-GGCACTGCTGATTGAAGACA-3′	5′-TGTCACCTGCACCTTCTCTG-3′
*MRP4*	5′-TCCTGATGATGGTGGCTGTA-3′	5′-ATGCAATTTCAGGGAGGTGA-3′
*MRP5*	5′-AGTTCTGTTTGTTACCCACCAGTT-3′	5′-ACCCTTGTCTTGTGACTTCTTCTG-3′
*MRP6*	5′-GCTCTATCCTCAGGAACTCGAAGAC-3′	5′-GCTTTCTCTGCATTCATAGCATTCT
*MRP7 (CFTR)*	5′-CTACTCTCCTTCGCCACATTTTC-3′	5′-TGGCTCAGAGAGGCCTTCTC-3′
*MRP8*	5′-CTCCTCAGGGATTTTCACCA-3′	5′-AGGACCAGGAACTGCTCTGA-3′
*MRP9*	5′-CCAAGACTGACACCCTGGTT-3′	5′-GACGTGATCGCAGRRGAGA-3′
*MRP10 (MRP7)*	5′-AAAGGGCACCCTGGATTACT-3′	5′-AATGCAAGTGGGCTCCTATG-3′
*P-gp*	5′-GCCAAAGCCAAAATATCAGC-3′	5′-TTCCAATGTGTTCGGCATTA-3′
*BCRP*	5′-CCCTGACATTCTGTCACAACA-3′	5′-GGGACAGGTATGTGAAAAGCA-3′
*MATE1*	5′-ATGCTGTTTCCCACCTCTTTG-3′	5′-CCGAGGCACGTTGTTTACTT-3′
*MATE2*	5′-TGGGGCATATTTTTACCAATG-3′	5′-GAACTCGCCCATAGACACAAC-3′
*OAT1*	5′-CAGCAACAAGAGCACCAGAA-3′	5′-TGGGTCACCATTTCCTCTTC-3′
*OAT2*	5′-CCCAAGGGACAAAAAGAACA-3′	5′-ATGAGACCAGTGGGTTGGAG-3′
*OAT3*	5′-TCCCAGAGGATCCCTCTACA-3′	5′-TGCCTGGCTAGGATCAGTCT-3′
*OCT1*	5′-TGGAGGCATGGTGAAATACA-3′	5′-GTCACCCACTTCCGTGATCT-3′
*OCT2*	5′-GATCCTGCCAAATTCTTCCA-3′	5′-TAGCCCACAGTTCCCCTATG-3′
*OCT3*	5′-ATGGTTGCTGAACCCAAAAC-3′	5′-CCCAGATCGTTAAACCCAGA-3′
*OCTN1*	5′-ACCCCATTTGGTGAAGTGAA-3′	5′-GGTTGGTTTGTAAAGCAAGGAC-3′
*OCTN2*	5′-ATTTGCCCTTCAGAATGCAC-3′	5′-GCAGACAATTGCCAGAGTGA-3′
*CNT1*	5′-AGGTCCTGCCCATCATTGTC-3′	5′-CAAGTAGGGCCGGATCAGTA-3′
*CNT2*	5′-AATGGGTGTTTGCAGGAGTC-3′	5′-GAAGACCTAGGCCCGAAAAC-3′
*ENT1*	5′-GCTGGGTCTGACCGTTGTAT-3′	5′-CTGTACAGGGTGCATGATGG-3′
*ENT2*	5′-AGCCTGCATGTGTGTACTGC-3′	5′-ACCACGGACCAGTCACTTTC-3′
*OATP1A2*	5′-GCATCAAGGGCAGATGATTT-3′	5′-GGCTGGGAAGTCAAGAGATG-3′
*OATP1B1*	5′-GCTGGGGCAGATAGTGAAAC-3′	5′-GGACCAGGAACTCCTCAAAA-3′
*OATP1B3*	5′-GAAAAGGTTGTTTAAAGGAATCTGG-3′	5′-CGAAATCATCAATGTAAGAAAGCC-3′
*OATP1C1*	5′-ACTCCCATTCAGCCTTTGGG-3′	5′-CAGAAAGGCACAGCTGCAAG-3′
*OATP2A1*	5′-TTCCAAAGCCACCTCATTTC-3′	5′-GGTTAGTTGCAGGGCATCAT-3′
*OATP2B1*	5′-GGCTTTGAGACTTTCCCACA-3′	5′-CTGGGAAACAAGAGGGATGA-3′
*OATP3A1*	5′-CAGGCCATGCTCTCCGAAA-3′	5′-CTGCTGCTCCAGGTACTTCC-3′
*OATP4A1*	5′-CTGCCAGCCAGAACACTACA-3′	5′-AGAAGGAGGGGCTTTCTCTG-3′
*OATP5A1*	5′-TCATGCTCCCCTACGGTACAG-3′	5′-GCTCACCTTTGTTTGGAGTGTTAG-3′
*OATP6A1*	5′-GGAGCCAGGATGAAGTCTCAA-3′	5′-GAACCTTATCAAGGCCTCTGGAAG-3′

## Supplementary Materials

The following are available online at https://www.mdpi.com/1999-4923/12/11/1005/s1. Figure S1. Relative mRNA expression of human TERT assessed by microarray represented as the log_2_-fold change. Figure S2. Relative mRNA expression of various SC marker genes described in Figure 3A assessed by microarray represented as the log_2_-fold change. Figure S3. (A) Relative mRNA expression of various xenobiotic efflux transporter genes assessed by microarray. (B) Relative mRNA expression of anion and cation transporter genes in the SLC22A family assessed by microarray. (C) Relative mRNA expression of organic anion transporting polypeptide transporter genes in the SLC21 family assessed by microarray. (D) Relative mRNA expression of concentrative and equilibrative nucleoside transporters assessed by microarray. All data are represented as the log_2_-fold change.
