# Supplementary material for: Generation of a hTERT-Immortalized Human Sertoli Cell Model to Study Transporter Dynamics at the Blood-Testis Barrier
Source: Pharmaceutics. 2020 Oct 22;12(11):1005. doi: 10.3390/pharmaceutics12111005 (PMC7690448; doi:10.3390/pharmaceutics12111005)
Supplement: Supplementary file 1 [file pharmaceutics-12-01005-s001.pdf]

Supplementary Materials: Generation of an hTERT-Immortalized Human Sertoli Cell Model to Study Transporter Dynamics at the Blood-Testis Barrier: Supplementary Materials

Raymond K. Hau, Siennah R. Miller, Stephen H. Wright and Nathan J. Cherrington

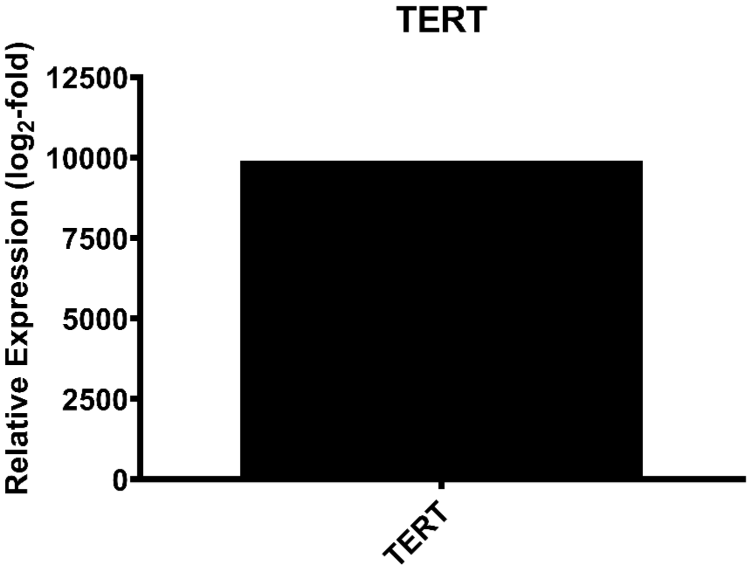

Figure S1. Relative mRNA expression of human TERT assessed by microarray represented as the log_2-fold change.

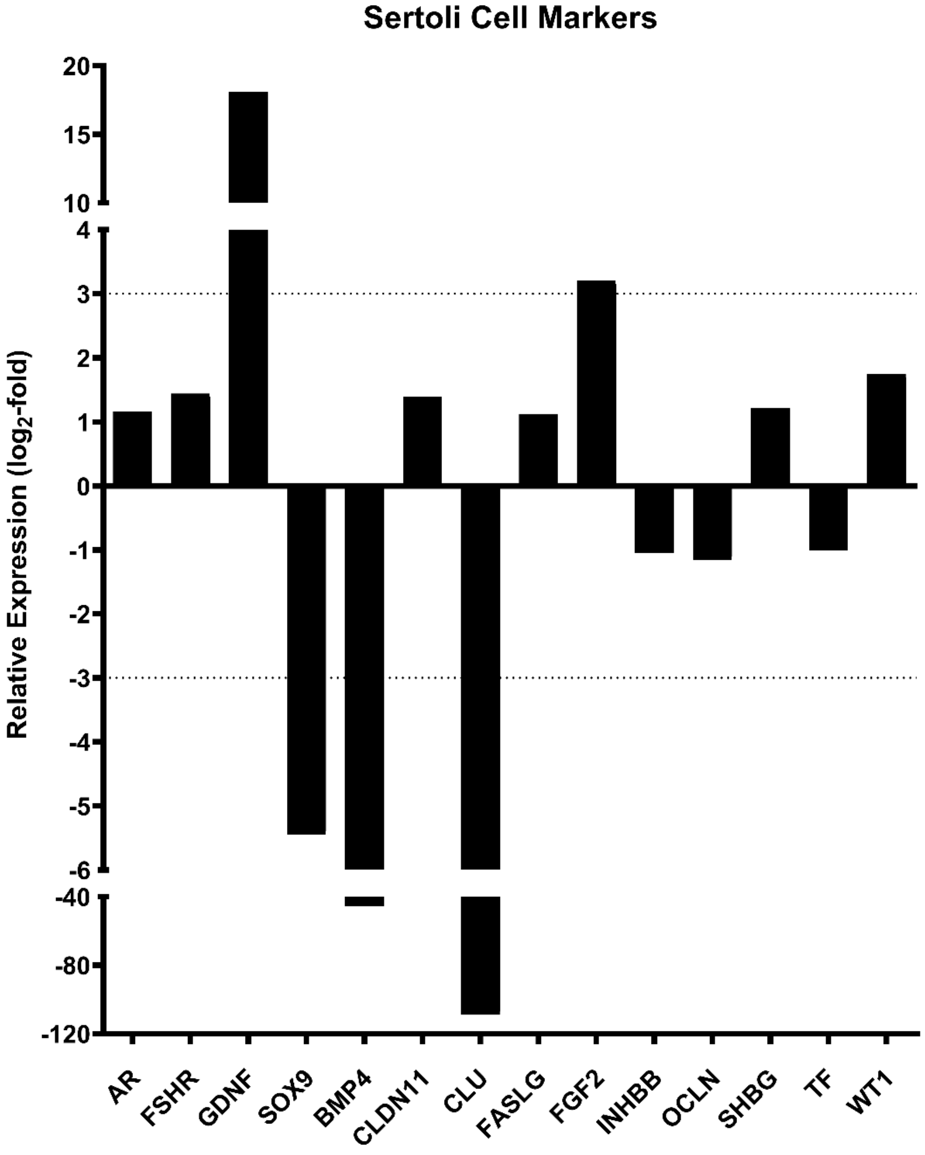

Figure S2. Relative mRNA expression of various human SC marker genes described in Figure 3A assessed by microarray represented as the $\log_2$-fold change.

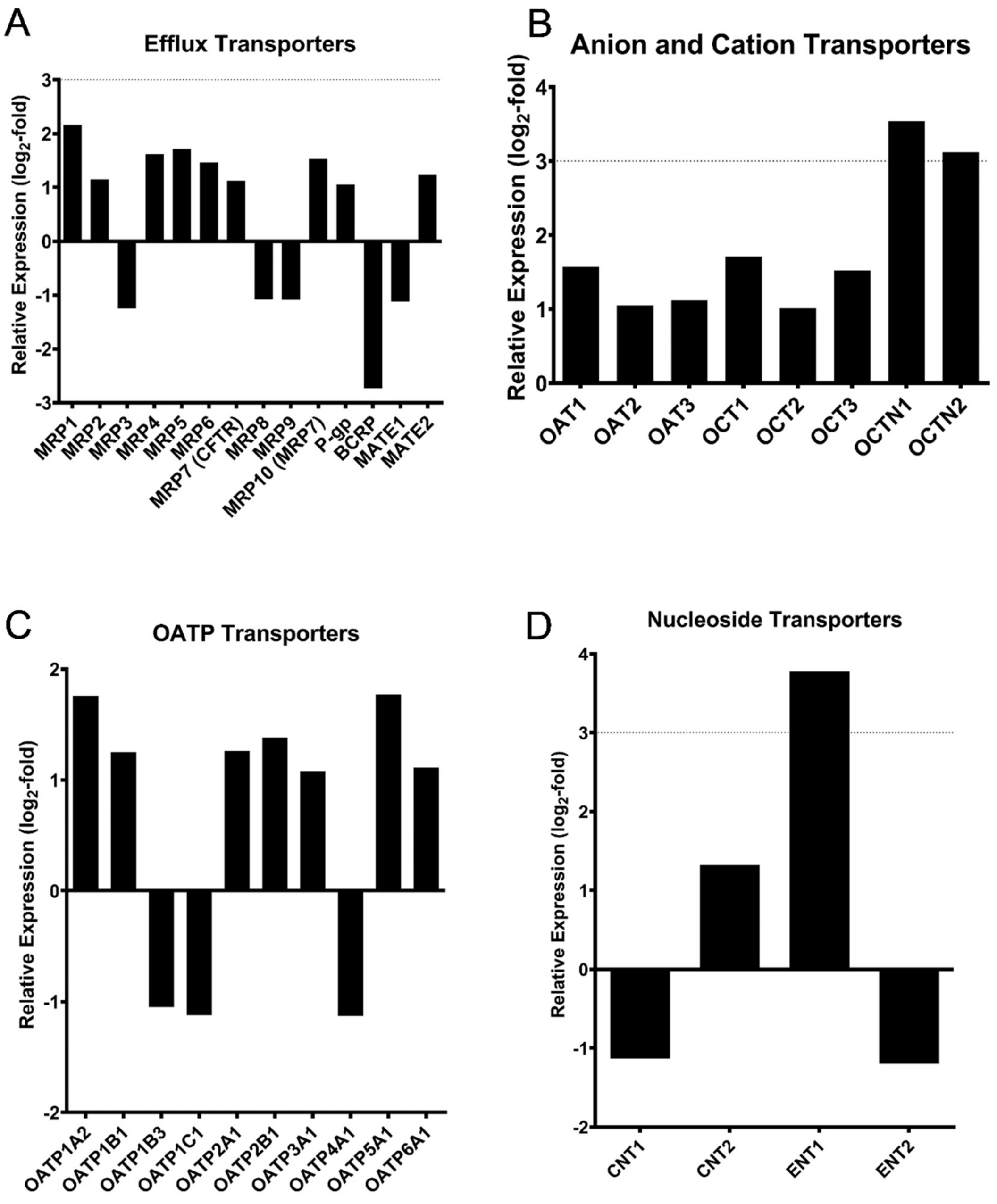

Figure S3. (**A**) Relative mRNA expression of various human xenobiotic efflux transporter genes assessed by microarray. (**B**) Relative mRNA expression of human anion and cation transporter genes in the SLC22A family assessed by microarray. (**C**) Relative mRNA expression of human organic anion transporting polypeptide transporter genes in the SLC21 family assessed by microarray. (**D**) Relative mRNA expression of human concentrative and equilibrative nucleoside transporters assessed by microarray. All data are represented as the log_2-fold change.
